# Neoadjuvant hormone therapy plus robot-assisted radical prostatectomy improves pathologic outcomes in high-risk prostate cancer: a retrospective cohort study

**DOI:** 10.1186/s12894-026-02153-x

**Published:** 2026-04-28

**Authors:** Junhong Fan, Shuang Liu, Ziyu Li, Yuting Xv, Teng Li, Shang Huang, Chunxiang Feng, Jiumin Liu, Dong Li, Fenglian Jiang

**Affiliations:** Department of Urology, Guangdong Provincial People’s Hospital (Guangdong Academy of Medical Sciences), Southern Medical University, Guangzhou, 510080 China

**Keywords:** High-risk prostate cancer, Neoadjuvant hormone therapy, Robot-assisted radical prostatectomy, Pathological complete response, Minimal residual disease, Positive surgical margin

## Abstract

**Background:**

To assess the impact of neoadjuvant hormone therapy (NHT) prior to robot-assisted radical prostatectomy (RP) in high-risk prostate cancer (HRPCa).

**Methods:**

We retrospectively analyzed 141 HRPCa patients who underwent robot-assisted RP from February 2022 to June 2025. According to whether NHT was administered preoperatively, patients were divided into NHT + RP group (*n* = 27) and RP-alone group (*n* = 114). The perioperative parameters and pathological outcomes were compared between groups.

**Results:**

At the baseline, the NHT + RP group had significantly higher fibrinogen (*p* = 0.003), AST (*p* = 0.034), PSA (*p* = 0.001), and PSA density (*p* < 0.001), indicating more aggressive disease. Even so, NHT + RP was associated with a trend toward reduced prostate volume (37.70 ± 31.14 vs. 46.07 ± 29.50 ml, *p* = 0.315) and improved pathological outcomes, including higher rates of pCR (18.5% vs. 0%, *p* < 0.001) and MRD (7.4% vs. 0%, *p* = 0.003), as well as a lower PSM rate (33.3% vs. 57.0%, *p* = 0.027). In multivariable analysis, NHT remained independently associated with reduced PSM (OR = 0.236, 95% CI: 0.068–0.820, *p* = 0.023).

**Conclusion:**

Preoperative NHT followed by robot-assisted RP in HRPCa improved key pathological outcomes, including higher pCR/MRD and lower PSM rates. Given the inherent limitations of the retrospective design, prospective studies are required to validate these findings and their long-term clinical benefit.

## Introduction

Prostate cancer (PCa) is the second most common male malignancy worldwide, and the incidence has shown a steadily increasing trend over recent years [1, 2]. In 2020, PCa accounted for 14.1% of all cancer cases (1,414,259 cases) and 6.8% of male cancer-related deaths (375,304 cases) [2]. In recent years, the proportion of newly diagnosed high-risk prostate cancer (HRPCa) patients in China has been significantly higher than that in foreign countries, approximately 15–20% [1, 3]. HRPCa refers to those with aggressive pathological grade, advanced T stage or high level of prostate-specific antigen (PSA). Compared with patients with low/moderate-risk PCa, patients with HRPCa have a higher risk of postoperative biochemical recurrence and metastasis progression. Approximately 20% of HRPCa patients experience biochemical recurrence within 1 year after surgery, and the cumulative rate reaches about 50% within 3–5 years [4, 5]. Even with intensified treatment including adjuvant or salvage radiotherapy and/or chemotherapy hormone therapy after surgery, the 15-year mortality rate remains above 30% [6]. HRPCa may have microscopic metastatic lesions that are not visible on conventional imaging, and it is often difficult to be completely eradicated by surgery alone, which may be the reason for its frequent postoperative biochemical recurrence and even disease progression. Therefore, it is urgent to explore more precise and effective treatment strategies to improve the long-term survival prognosis and quality of life of patients with HRPCa.

At present, there is no consensus on the optimal treatment strategies for patients with HRPCa. The recommended treatment model is a comprehensive approach based on radical prostatectomy (RP). Traditional androgen deprivation therapy (ADT) has not been recommended by guidelines as it fails to improve the long-term survival of patients [7]. Although neoadjuvant hormone therapy (NHT) may carry potential risks such as ineffectiveness, delaying the surgery, increasing surgical complications, and over-treatment, it remains a treatment strategy worth active exploration in HRPCa in order to avoid or reduce the high risk of recurrence after local treatment. In recent years, with the continuous development of new drugs, new endocrine drugs such as abiraterone and enzalutamide have achieved certain results in NHT [8]. NHT during the perioperative period of PCa has received increasing attention.

In this study, we retrospectively evaluated the efficacy and safety of NHT combined with robot-assisted RP versus robot-assisted RP alone in patients with HRPCa, thereby providing more clinical experience for NHT combined with robot-assisted RP. Our aim is to provide some meaningful guidance for clinicians in optimizing perioperative treatment strategies for patients with HRPCa.

## Methods

### Study design and participants

This study was approved by the Institutional Research Ethics Committee of Guangdong Provincial People’s Hospital and in accordance with the Declaration of Helsinki, KY-Z-2022-354-01. Due to the retrospective nature of the study and complete anonymization of data, the requirement for informed consent was waived by the ethics committee.

Individuals were eligible for inclusion if patients’ diagnosis of PCa by biopsy and eligible for RP, biochemical, pathological and imaging findings indicating primary tumor staging for HRPCa. Exclusion criteria were: (1) small cell, neuroendocrine, or sarcomatoid features in pathology; (2) radiological evidence of metastasis of viscera or bone; (3) previously treated with chemotherapy and/or radiotherapy for PCa; (4) severe or uncontrollable chronic or infectious disease; (5) other malignant tumors within 5 years; (6) the incomplete medical records. Finally, a total of 141 HRPCa patients who underwent robotic-assisted RP at our center between February 2022 and June 2025 were identified and their clinical data were reviewed. Patients were divided into two groups: the RP group (114 cases) and the NHT + RP group (27 cases), based on whether NHT was administered preoperatively.

### Treatments

Patients in the NHT + RP group received preoperative treatment with apalutamide (240 mg/day) or abiraterone acetate (1000 mg/day) plus prednisone (5 mg/day) for an average duration of 3.6 months (3–6 months), along with concurrent ADT via subcutaneous goserelin or intramuscular triptorelin injections. All RPs were performed by one experienced urologist.

### The definition of HRPCa

According to the risk-stratification models described in the European Association of Urology (EAU) and National Comprehensive Cancer Network (NCCN) prostate cancer guidelines, risk groups are primarily defined on the basis of serum PSA level, Gleason score (GS) on biopsy, and clinical TNM stage [9]. In this study, HRPCa encompasses organ-confined high- or very high-risk disease as well as locally advanced PCa. HRPCa is specifically defined by the presence of at least one of the following criteria in the absence of distant metastasis: PSA > 20 ng/mL, GS ≥ 8 on biopsy, or clinical stage ≥ cT2c. Locally advanced disease generally refers to tumor with regional spread limited to pelvic lymph node metastasis [10, 11].

### Clinical data and laboratory assessment

The clinical characteristics and blood chemistry assays, including age, body mass index (BMI), hypertension, diabetes, coronary heart disease (CHD), fibrinogen (FIB), aspartate amino transferase (AST), alanine aminotransferase (ALT), gamma-glutamyl transferase (GGT), alkaline phosphatase (ALP), were collected through medical records. Blood was collected after overnight fast. We also evaluated the prostate volume (PV), serum PSA values, PSA density (PSAD), GS and clinical TNM stage at diagnosis. Perioperative parameters included operation time and hemoglobin (Hb) decrease. Oncologic outcomes included pathological T stage, pathological GS, positive surgical margins (PSM), intraductal carcinoma of the prostate (IDC-P), pathological complete response (pCR), minimal residual disease (MRD).

IDC-P was defined as lumen-spanning proliferation of carcinoma cells distending antecedent ducts or glands. PV is calculated according to the formula 0.52 * anterior diameter * lateral diameter * superior diameter. PSAD is calculated by dividing the PSA value at the initial diagnosis by the PV. A favorable pathological response was defined as the presence of a pCR or MRD in the RP specimen; pCR is defined as the absence of morphologically identifiable carcinoma in the prostatectomy specimen, and MRD is defined as the maximum size of the residual tumor burden in the prostatectomy specimen being ≤ 5 mm [3, 12].

### Statistical analysis

Statistical analysis was performed using the SPSS software version 20.0. Continuous variables were expressed as mean ± SD. Kolmogorov–Smirnov criterion was applied to test for normal distribution. Differences were assessed by the Student’s t-test in normal variables and by the Mann–Whitney U-test in nonnormal variables. Categorical variables were analyzed for statistical significance using chi-square test or Fisher exact test. Multivariable logistic regression analysis was used to identify the independent factors associated with PSM. Variables included in the model were selected based on clinical relevance and baseline differences, including NHT, PSA, PSAD, pathological Gleason score (≥ 8 vs. < 8), and pathological T stage (≥ T3 vs. < T3). Odds ratios (ORs) with 95% confidence intervals (CIs) were calculated. *p* < 0.05 was considered to be statistically significant in overall comparison.

## Result

A total of 141 HRPCa patients were included in this retrospective study. Patients were divided into two groups based on whether they received NHT preoperatively. One group (*n* = 27) received NHT combined with robot-assisted RP, while another group (*n* = 114) underwent robot-assisted RP alone. Patients in the NHT + RP group demonstrated favorable tolerance to the novel hormonal agents and goserelin/triptorelin. Only one patient experienced a mild rash. No patients experienced severe hepatic, cardiac or renal toxicity, fractures, gastrointestinal reactions, or neurological involvement.

The overview patients’ demographics and characteristics are listed in Table 1. The mean age of the NHT + RP group was a little older than the RP group (69.85 ± 5.65 vs. 68.11 ± 7.20 years, *p =* 0.244). Compared to the RP only group, patients in NHT + RP group had significantly higher FIB (3.71 ± 0.89 vs. 3.21 ± 0.72 g/L, *p* = 0.003), AST (26.41 ± 11.93 vs. 21.13 ± 6.50 U/L, *p* = 0.034) and PSA (57.74 ± 57.32 vs. 19.61 ± 21.42 ng/ml, *p* = 0.001) levels in the laboratory blood test results. There were no significant differences in regards to BMI, hypertension, diabetes, CHD, ALT, GGTand ALPbetween these two groups (*p* > 0.05).

**Table 1:** Preoperative general characteristics of patients

Variable	RP	NHT + RP	*P* value
No. of patients	114	27	
Clinical data			
Age (years)	68.11 ± 7.20	69.85 ± 5.65	0.244
BMI (kg/m^2^)	24.17 ± 2.85	24.02 ± 2.82	0.814
Hypertension, n (%)	52(45.6)	13(48.1)	0.812
Diabetes, n (%)	21(18.4)	4(14.8)	0.659
CHD, n (%)	11(9.6)	4(14.8)	0.434
Laboratory data			
FIB (g/L)	3.21 ± 0.72	3.71 ± 0.89	0.003
AST (U/L)	21.13 ± 6.50	26.41 ± 11.93	0.034
ALT (U/L)	21.25 ± 11.48	24.56 ± 15.65	0.213
GGT (U/L)	32.66 ± 21.29	32.76 ± 25.99	0.984
ALP (U/L)	69.11 ± 20.61	80.32 ± 30.15	0.088
PSA (ng/ml)	19.61 ± 21.42	57.74 ± 57.32	0.001

Variables are presenting as n (range), n (%) or mean ± s.d

*RP* Radical prostatectomy, *NHT* Neoadjuvant hormone therapy, *BMI* Body mass index, *CHD* Coronary heart disease, *FIB* Fibrinogen, *AST* Aspartate amino transferase, *ALT* Alanine aminotransferase, *GGT* Gamma-glutamyl transferase, *ALP* Alkaline phosphatase, *PSA* Prostate-specific antigen

Perioperative pathological and oncologic outcomes are summarized in Table 2. Compared to the RP only group, patients in NHT + RP group had significantly higher PSAD (1.61 ± 1.33 vs. 0.53 ± 0.51 ng/ml^2^, *p*<0.001) level. Pathological T stage distributions showed a significantly higher proportion of pCR and MRD in the NHT + RP group compared to the RP-only group (18.5% vs. 0%, *p*<0.001 and 7.4% vs. 0%, *p* = 0.003, respectively), 5 patients (18.5%) reached pCR and 2 patients (7.4%) had MRD (Table 2). PSM was observed in 9 patients in the NHT + RP group and 65 in the RP-only group (33.3% vs. 57.0%, *p* = 0.027). The distribution of pathological Gleason scores was similar between the two groups, approximately half of the patients in both groups had scores ranging from 8 to 10. Similarly, the Clinical T stage, biopsy Gleason score, imaging PV, operative time, hemoglobin decrease, IDC-P, and pathological PV were similar between the two group (*p*>0.05). No clinically documented cases of deep vein thrombosis (DVT) or pulmonary embolism (PE) were identified in either group based on retrospective review of medical and nursing records.

**Table 2:** Postoperative, pathological and oncologic parameters

Variable	RP	NHT + RP	*P* value
No. of patients	114	27	
Imaging and pathological data pre-RP			
Clinical T stage at diagnosis			0.053
cT2, n (%)	95 (83.3)	18 (67.7)	
cT3a, n (%)	13 (11.4)	4 (14.8)	
cT3b, n (%)	6 (5.3)	5 (18.5)	
Biopsy Gleason score ≥ 8, n (%)	50 (43.9)	17 (63.0)	0.074
Imaging PV (ml)	38.25 ± 18.14	46.07 ± 29.50	0.149
PSAD (ng/ml^2^)	0.53 ± 0.51	1.61 ± 1.33	<0.001
Pathological and oncological data post-RP			
Operation time (min)	233.14 ± 61.84	218.92 ± 51.13	0.287
Hemoglobin decrease (g/L)	14.61 ± 9.11	14.77 ± 8.41	0.937
Pathological PV (ml)	42.97 ± 22.08	37.70 ± 31.14	0.308
Pathological Gleason score ≥ 8, n (%)	54 (47.4)	14 (51.9)	0.675
IDC-P, n (%)	11 (9.6)	1 (3.7)	0.319
PSM, n (%)	65 (57.0)	9 (33.3)	0.027
Pathological T stage			<0.001
pT2, n (%)	69 (60.5)	7 (25.9)	
pT3a, n (%)	20 (17.5)	6 (22.2)	
pT3b, n (%)	25 (21.9)	7 (25.9)	
pCR/MRD, n (%)	0	7 (25.9)	
pCR, n (%)	0	5 (18.5)	<0.001
MRD, n (%)	0	2 (7.4)	0.003

*RP* Radical prostatectomy, *NHT* Neoadjuvant hormone therapy, *PV* Prostate volume, *PSAD* PSA density, *IDC* Intraductal carcinoma of the prostate, *PSM* Positive surgical margins, *pCR* pathological complete response, *MRD* Minimal residual disease

As shown in Table 3. In the NHT + RP group, the PV showed a decreasing trend after NHT treatment (46.07 ± 29.50 vs. 37.70 ± 31.14 ml, *p* = 0.315), with a median percentage reduction of 32.17% (IQR: 7.87–71.47%). After NHT treatment, the number of patients with a pathological Gleason score of ≥ 8 slightly decreased from 17 to 14 (63.0% vs. 51.9%, *p* = 0.409), and 25.9% of the patients experienced a pathological downgrade.

**Table 3:** The data of patients in the NHT + RP group before and after receiving NHT treatment

Variable	pre-NHT	post-NHT	*P* value
No. of patients	27	27	
PV (ml)	46.07 ± 29.50	37.70 ± 31.14	0.315
Pathological Gleason score ≥ 8, n (%)	17 (63.0)	14 (51.9)	0.409

*NHT* Neoadjuvant hormone therapy, *PV* Prostate volume

Multivariable logistic regression analysis was performed to adjust for baseline differences (Table 4). NHT remained independently associated with a lower risk of PSM (OR = 0.236, 95% CI: 0.068–0.820, *p* = 0.023). Pathological T stage ≥T3 was identified as an independent risk factor for increased PSM (OR = 12.935, 95% CI: 4.771–35.067, *p* < 0.001), while PSA, PSAD, and pathological Gleason score were not independently associated with PSM.

**Table 4:** Logistic regression analysis for factors associated with the PSM

Variable	Multivariate		
	OR	95%CI	*P* value
NHT	0.236	0.068–0.820	0.023
PSA	0.993	0.974–1.012	0.466
PSAD	1.141	0.460–2.827	0.776
Pathological Gleason score ≥ 8	1.008	0.421–2.412	0.986
Pathological T stage ≥ 3	12.935	4.771–35.067	<0.001

*NHT* Neoadjuvant hormone therapy, *PSAD* PSA density, *PSM* Positive surgical margins, *PSA* Prostate-specific antigen

## Discussion

In clinical practice, the management of high-risk or locally advanced PCa remains challenging, and results with RP alone are unsatisfactory. Therefore, it is necessary to explore a multi-modal comprehensive treatment strategy based on local treatment (surgery or radiotherapy) in order to enhance the long-term efficacy and improve the prognosis of patients. NHT is defined as induction treatment administered before final local therapy, it aims to decrease the primary tumor burden and eradicate possible microscopic metastatic lesions. Such neoadjuvant strategies have been successfully performed in breast, colorectal and lung cancers [13–15]. NHT incorporating novel androgen receptor pathway inhibitors (ARPIs) before local therapy for PCa has attracted growing interest, while the exact clinical benefits and optimal use remain further investigation. In this retrospective study, we found that preoperative NHT combined with ARPIs significantly improved pathological outcome, including higher percentages of pCR (18.5% vs. 0%, *p*<0.001), MRD (7.4% vs. 0%, *p* = 0.003) and lower PSM (33.3% vs. 57.0%, *p* = 0.027).

FIB and AST, although non-specific biomarkers, have been associated with adverse oncological features and poorer prognosis in PCa [16–21]. In our cohort, both FIB and AST were significantly elevated in the NHT + RP group (*p* < 0.05), which may reflect a higher systemic inflammatory and metabolic burden. Previous studies have also demonstrated a positive correlation between FIB and PSA levels [22, 23], supporting its association with tumor burden. Taken together, the concurrent elevation of these parameters suggests that patients receiving neoadjuvant therapy had a more unfavorable baseline condition, reflecting potential selection bias rather than a simple background difference.

In contrast, PSA and PSAD are more direct indicators of the tumor burden and local invasiveness of the prostate. The initial PSAD before treatment has been repeatedly demonstrated to be associated with the adverse pathological features at RP and the increased biochemical recurrence risk, especially in HRPCa [24, 25]. In clinical practice, the PSAD thresholds between 0.15 and 0.35 ng/mL^2^ are usually regarded as having substantial clinical significance for the diagnosis of PCa. Moreover, previous studies have confirmed that PSAD is another independent prognostic indicate in addition to PSA [24–26]. A higher PSAD (1.61 ± 1.33 vs. 0.53 ± 0.51 ng/ml^2^, *p*<0.001) is consistent with the larger or more aggressive tumors, which would theoretically lead to higher PSM rate and lower local control with RP alone.

Systematic treatment before surgery has the potential to achieve better oncological and functional outcomes, and it strengthens the biological basis for the use of neoadjuvant therapy in specific patients [27]. In this study, NHT followed by RP achieved a substantially higher rate of deep pathologic response, including significantly higher pCR (18.5% vs. 0%, *p*<0.001), MRD (7.4% vs. 0%, *p* = 0.003) and lower PSM (33.3% vs. 57.0%, *p* = 0.027) compared to RP alone. These findings are consistent with results of several recent studies about neoadjuvant treatment strategies for HRPCa, which revealed that 15–30% of patients achieved pathologic downstaging after 4–6 months of neoadjuvant therapy [12, 28]. Such deep pathologic responses (pCR/MRD) are increasingly recognized as clinically relevant intermediate endpoints for long-term outcome. Lower residual cancer burden after neoadjuvant ARPI-based therapy correlates with improved biochemical control and metastasis free survival [28, 29].

The lower PSM rate observed in the NHT + RP group provides anatomical support for the biological effects of neoadjuvant therapy. Previous studies have demonstrated that neoadjuvant androgen deprivation can decrease pathological stage and PSM rates, although without clear long-term survival benefit [3, 12]. Mechanistically, NHT may decrease tumor volume, limit extraprostatic extension, and modify the tumor–stromal interface, thereby facilitating more complete resection within an oncologically appropriate plane and reducing residual microscopic disease at the margin [1, 3, 12]. This effect is particularly relevant in HRPCa, where high tumor burden and frequent apical or posterolateral involvement contribute to increased surgical difficulty and higher baseline PSM risk. Importantly, to address potential confounding due to baseline imbalance, multivariable analysis was performed (Table 4), which confirmed that NHT remained independently associated with reduced PSM after adjustment. This finding suggests that the observed benefit was not solely driven by differences in baseline characteristics, thereby strengthening the robustness of the results despite the retrospective design. Taken together, despite the presence of more adverse baseline features in the NHT + RP group (e.g., higher PSA and PSAD), the consistent reduction in PSM supports the role of neoadjuvant intensification in improving local disease control in HRPCa.

IDC-P provides an additional dimension to biological interpretation. IDC-P is significantly associated with higher Gleason grade [30], increased probability of lymph node metastasis [31], advanced disease stage [30], as well as elevated risks of progression and cancer-specific mortality after RP [32]. Several studies have identified IDC-P as an independent adverse prognostic factor even after adjustment for standard clinicopathological variables [30–32]. In our study, the incidence of IDC-P (3.7% vs. 9.6%, *p* = 0.319) in the NHT + RP group was obviously lower without statistical difference. This is consistent with the recent biomarker-directed neoadjuvant studies where the IDC-P and cribriform morphology are more common in non-responders and residual high-volume lesions [33, 34]. It indicates that these lesions may represent morphological changes associated with intrinsic treatment resistance. Therefore, although the pCR/MRD rate in the NHT + RP group was significantly higher, the lack of statistically significant IDC-P reduction might reflect the limited sample size and biological reality, that short-term NHT is more effective in reducing the volume of traditional acinar carcinoma than in eradicating the invasive intraductal components.

In the NHT + RP group, the higher pCR/MRD, lower PSM, and the non-significant but directionally favorable trend of IDC-P indicate that NHT can partially rebalance the adverse baseline risk of HRPCa patients at the local pathological level. These data further confirm the view that the deep pathological response observed after neoadjuvant ARPI-based therapy is not merely a special histological change, but rather a comprehensive indicator that include RP, tumor biological characteristics, and the removal of lesions at the microstructural level. In addition, the persistence of IDC-P and heterogeneous depth of pathological response emphasize the need for molecular level detection and longer treatment duration in future neoadjuvant strategies.

While this study provides valuable insights into the efficacy of NHT combined with RP in HRPCa, several limitations should be acknowledged. First, the retrospective design inherently introduces potential selection bias, as treatment allocation was based on physician discretion and clinical judgment, and therefore cannot be fully controlled. Second, the relatively small sample size, particularly in the NHT group, may limit statistical power and generalizability, and precluded further stratified analyses (e.g., by specific NHT regimens), which may introduce heterogeneity in treatment effects. Third, the absence of long-term follow-up data limits the evaluation of clinically meaningful outcomes such as biochemical recurrence, metastasis-free survival, and overall survival, and thus the findings should be interpreted primarily in the context of short-term pathological endpoints. Finally, due to the retrospective nature of data collection and the lack of routine screening protocols, postoperative complications—especially asymptomatic events such as thromboembolism—may have been underdiagnosed or underreported. Despite these limitations, the consistency of findings across multiple pathological endpoints and the results of multivariable analysis support the robustness of the observed association.

## Conclusion

In conclusion, NHT combined with robot-assisted RP was associated with improved pathological outcomes in patients with high-risk prostate cancer, including higher rates of pCR/MRD and a lower rate of positive surgical margins. These findings suggest a potential benefit of neoadjuvant intensification in enhancing local disease control. However, given the retrospective design, baseline imbalances, limited sample size, and lack of long-term follow-up data, these results should be interpreted with caution. Importantly, the observed pathological improvements represent early indicators of treatment activity rather than definitive evidence of long-term oncologic benefit. Further prospective studies with larger cohorts and extended follow-up are warranted to determine whether these pathological responses translate into durable clinical outcomes.

## Data Availability

The datasets used and/or analysed during the current study are available from the corresponding author on reasonable request.

## Abbreviations

PCa: Prostate cancer
HRPCa: High-risk prostate cancer
PSA: Prostate-specific antigen
ADT: Androgen deprivation therapy
NHT: Neoadjuvant hormone therapy
RP: Radical prostatectomy
EAU: European Association of Urology
NCCN: National Comprehensive Cancer Network
GS: Gleason score
CHD: Coronary heart disease
FIB: Fibrinogen
AST: Aspartate amino transferase
ALT: Alanine aminotransferase
GGT: Gamma-glutamyl transferase
ALP: Alkaline phosphatase
PV: Prostate volume
PSAD: PSA density
Hb: Hemoglobin
PSM: Positive surgical margins
IDC-P: Intraductal carcinoma of the prostate
pCR: pathological complete response
MRD: Minimal residual disease
